# Health-Related Quality of Life and Associated Factors Before and After Coronary Artery Bypass Grafting: A Multicentre Prospective Study in Cyprus

**DOI:** 10.1093/icvts/ivag126

**Published:** 2026-04-24

**Authors:** Kyriakos Alexandrou, Nicos Middleton, Maria Kyranou, Pavlos Sarafis

**Affiliations:** Nursing Department, Cyprus University of Technology, Limassol 3036, Cyprus; Nursing Department, Cyprus University of Technology, Limassol 3036, Cyprus; Nursing Department, Cyprus University of Technology, Limassol 3036, Cyprus; Department of Nursing, University of Thessaly, Volos, Greece

**Keywords:** coronary artery bypass grafting, health-related quality of life, arrhythmias, gender, quality of life

## Abstract

**Objectives:**

Coronary artery bypass grafting (CABG) improves survival in coronary artery disease (CAD), but its impact on health-related quality of life (HRQOL) remains variable. This study aimed to evaluate changes in HRQOL and associated factors before and after CABG.

**Methods:**

This prospective multicentre study, conducted across 3 hospitals in Cyprus, included patients undergoing elective CABG. Health-related quality of life was assessed using the MacNew questionnaire preoperatively and at 3 and 6 months postoperatively. Associations with demographic and clinical factors, including age, gender, postoperative arrhythmias, left ventricular ejection fraction, and type of surgery, were examined. Non-parametric methods were applied due to non-normal data distribution.

**Results:**

Health-related quality of life declined at 3 months compared with baseline (5.03 ± 1.02 vs 4.75 ± 1.00; *P* = .015), but improved significantly at 6 months (5.99 ± 0.67), exceeding preoperative levels. Male gender was associated with higher HRQOL scores at both follow-up time points (*P* = .011 and *P* = .009). No significant differences were observed by age, postoperative arrhythmia, left ventricular ejection fraction, or type of surgery.

**Conclusions:**

Health-related quality of life declined early after CABG but improved significantly by 6 months, exceeding baseline levels. Male gender was associated with higher HRQOL scores. These findings support the value of incorporating HRQOL assessment into routine postoperative care.

**Trial registration:**

Thai Clinical Trials Registry (TCTR), ID: TCTR202247. URL: https://www.thaiclinicaltrials.org/

## Graphical Abstract

**Figure:**
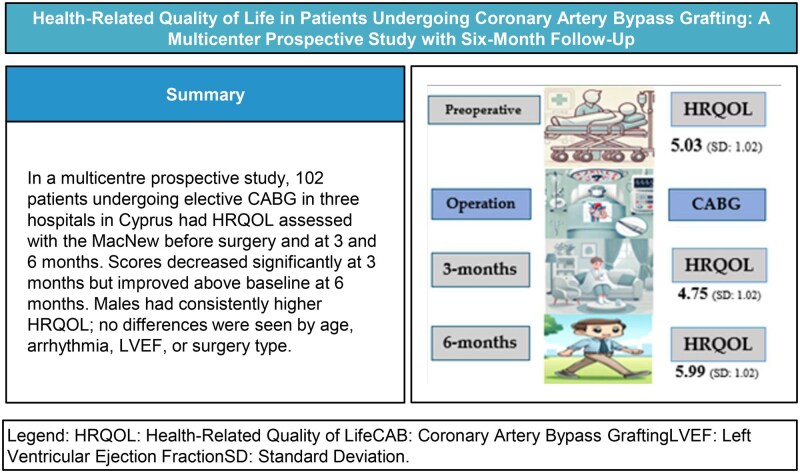

## INTRODUCTION

Cardiovascular diseases remain leading cause of death worldwide, with coronary artery disease (CAD) being the most prevalent.^1^ Coronary artery bypass grafting (CABG), performed since the 1960s, remains one of the most common surgical treatments, with about 400 000 procedures annually in the United States.^2–4^ Beyond survival and reduction of cardiac events, current patient-centred care highlights health-related quality of life (HRQOL) as an essential outcome.^5^

Health-related quality of life encompasses physical, emotional, and social functioning, but findings on its improvement after CABG are inconsistent, varying with demographic, clinical, and procedural characteristics.^6–14^

This prospective multicentre study evaluated HRQOL before and 6 months after CABG in Cyprus and explored demographic and clinical factors influencing postoperative recovery.

## METHODS

### Study design

This prospective multicentre study evaluated HRQOL before and 6 months after elective CABG in Cyprus. Patients were enrolled between September 2022 and April 2023 from 2 private and 1 public hospital, representing 3 of the 4 national CABG centres. Health-related quality of life was assessed preoperatively (within 1 week before surgery) and at 3 and 6 months postoperatively using the MacNew Heart Disease questionnaire. Follow-up assessments were performed by trained research staff via standardized telephone interviews.

### Study population

During the study period, 102 patients scheduled for elective CABG were enrolled, including 67 isolated CABG and 35 combined CABG with valve surgery. Eligible participants were adults (≥18 years) able to provide preoperative HRQOL data; emergency cases and patients unable to participate (eg, intubated or critically ill) were excluded. All underwent elective surgery for angiographically confirmed multivessel or left main CAD suitable for surgical revascularization. Data on preoperative functional status (eg, NYHA class) were not systematically recorded, as the study focused on HRQOL outcomes.

In combined cases, valve intervention was indicated for concomitant valvular disease requiring repair or replacement. Convenience sampling was applied due to recruitment constraints. Of 111 invited patients, 102 (91.9%) consented (80 men, 22 women). Five patients were not followed up (4 lost, 1 death), leaving 97 for final analysis. The single patient who died during follow-up was excluded from HRQOL analysis, as the MacNew questionnaire requires patient self-reporting.

### Data collection and tools

Data were collected preoperatively (within 1 week before surgery) and at 3 and 6 months postoperatively. Baseline assessments were performed during hospital admission; follow-ups were conducted via standardized telephone interviews by trained staff. Demographic and clinical variables (age, sex, postoperative arrhythmias, left ventricular ejection fraction [LVEF], and surgery type) were obtained from hospital records.

Health-related quality of life was assessed using the validated Greek version of the MacNew questionnaire,^12^^,^^15^ administered with permission from its developers. The instrument contains 27 items across emotional, physical, and social domains, scored on a 7-point Likert scale (1 = poorest, 7 = best). Domain and overall scores were calculated as the mean of completed items; scores ≥5 were considered indicative of satisfactory HRQOL. A change ≥0.5 was considered clinically significant.^12^^,^^15^^,^^16^ Missing responses for sensitive items (eg, sexual activity) were excluded without invalidating domain scores.^17^^,^^18^

### Statistical analysis

A priori power analysis was performed for the primary within-subject comparison of HRQOL between preoperative and postoperative assessments. A total of 28 participants per time point (total = 84) was required to achieve 90% power at α  =  0.05 for an expected medium-to-large standardized effect size (Cohen’s d = 0.8), based on previous CABG HRQOL studies.^19–21^ The minimal clinically important difference (MCID = 0.5), as defined for the MacNew questionnaire, was used to interpret the clinical relevance of observed changes rather than to inform the a priori sample size calculation. To account for an anticipated 20% attrition rate, the recruitment target was increased to 102 patients. Therefore, the study was powered for the primary HRQOL change over time, but not for multivariable modelling or smaller subgroup effects.

Missing data comprised <5% of the total sample (5 patients) and were assumed to be missing at random. Analyses were performed on complete cases (*n* = 97). MacNew HRQOL scores were calculated according to the manual. As data were non-normally distributed (Shapiro-Wilk test), non-parametric tests were applied: Wilcoxon signed-rank for within-subject changes, Kruskal-Wallis for age and LVEF, and Mann-Whitney U for gender, postoperative arrhythmia, and surgery type. All analyses were 2-tailed, with statistical significance set at *P* < .05, and were performed using Statistical Package for the Social Sciences (SPSS) v28.0 (IBM Corp., Armonk, NY, USA).

Gender-based comparisons were pre-specified as exploratory, given evidence of sex-related HRQOL differences after CABG. Although repeated-measures models (eg, mixed-effects ANOVA) are suitable for longitudinal data, they were not applied due to the non-normal distribution and modest sample size, which may have limited model stability. Effect sizes (r = Z/√N) were calculated for non-parametric tests. No formal correction for multiple comparisons was applied, as analyses were exploratory and hypothesis-generating.

### Ethical issues

The study was conducted in accordance with the principles of the Declaration of Helsinki and the WMA Declaration of Taipei on ethical considerations regarding health databases and biobanks. The study protocol was reviewed and approved by the Cyprus National Bioethics Committee (approval no. 2021.01.153) and the State Health Services Organization.

The study was registered with the Thai Clinical Trials Registry (TCTR), ID: TCTR202247; Date of registration: January 3, 2026. Registration details are available at: https://www.thaiclinicaltrials.org/

Written informed consent was obtained from all participants prior to inclusion in the study.

All data were collected and stored in accordance with applicable data protection regulations, with measures in place to ensure participant confidentiality.

Permission to use the validated Greek version of the MacNew questionnaire was granted by its developers.

## RESULTS

Ninety-seven patients were included in the final analysis. The cohort consisted predominantly of men (78%) with a mean age of 67 years and preserved left ventricular function (mean LVEF 49%). Most underwent isolated CABG (66%), while one-third had combined CABG with valve surgery. Six-month follow-up was achieved in 95% of cases, with in-hospital survival of 99%. Postoperative complications were uncommon, mainly atrial fibrillation (AF) (21%) and isolated cases of acute renal failure requiring dialysis (3%). Median ICU and hospital stays were 1 and 6 days, respectively. All procedures were performed on-pump with complete revascularization achieved. The median aortic cross-clamp time was 44.5 minutes (interquartile range [IQR]: 41.5-54.0). Baseline characteristics are shown in **Table 1**.

**Table 1.** Demographic and Clinical Characteristics of Participants

Participants—gender		
	Male, *n* = 80	Female, *n* = 22
	*Ν* (%)	*N* (%)
Age (years)		
≤59	15 (18.7)	3 (13.6)
60-69	31 (38.8)	8 (36.4)
≥70	34 (42.5)	11 (50)
Hypertension		
Yes	48 (60)	13 (59.1)
Non	32 (40)	9 (40.9)
Dyslipidaemia		
Yes	52 (65)	16 (72.7)
Non	28 (35)	6 (27.3)
Diabetes		
Yes	34 (42.5)	12 (54.6)
Non	46 (57.5)	10 (45.4)
LVEF		
LVEF ≤40%	12 (15.0)	7 (31.8)
LVEF 41%-50%	36 (45.0)	9 (40.9)
LVEF >50%	32 (40.0)	6 (27.3)
Type of surgery		
CABG	56 (70.0)	11 (50.0)
CABG+AV repair	5 (6.3)	5 (22.7)
CABG+AV replacement	9 (11.3)	2 (9.1)
CABG+MV repair	2 (2.4)	1 (4.5)
CABG+MV replacement	8 (10.0)	3 (13.6)

Abbreviations: AV, aortic valve; CABG, coronary artery bypass grafting; LVEF, left ventricular ejection fraction; MV, mitral valve.

### HRQOL and its domains

Health-related quality of life, assessed with the MacNew questionnaire in 102 patients, improved significantly 6 months after CABG (mean 5.03 ± 1.02 preoperatively vs 5.99 ± 0.67; *P* < .001), exceeding the minimal clinically important difference (MCID = 0.5). At 3 months, scores declined transiently (mean 4.75 ± 1.00; *P* = .015) without clinical significance but showed clear recovery by 6 months, supporting a sustained improvement after CABG.

All 3 MacNew domains followed similar trends. Emotional scores increased from 4.98 ± 0.85 preoperatively to 5.82 ± 0.54 at 6 months (*P* < .001). Physical scores declined from 5.04 ± 1.33 to 4.53 ± 1.26 at 3 months (*P* < .001) but improved to 6.18 ± 0.87 by 6 months (*P* < .001).

**Figure 1.**
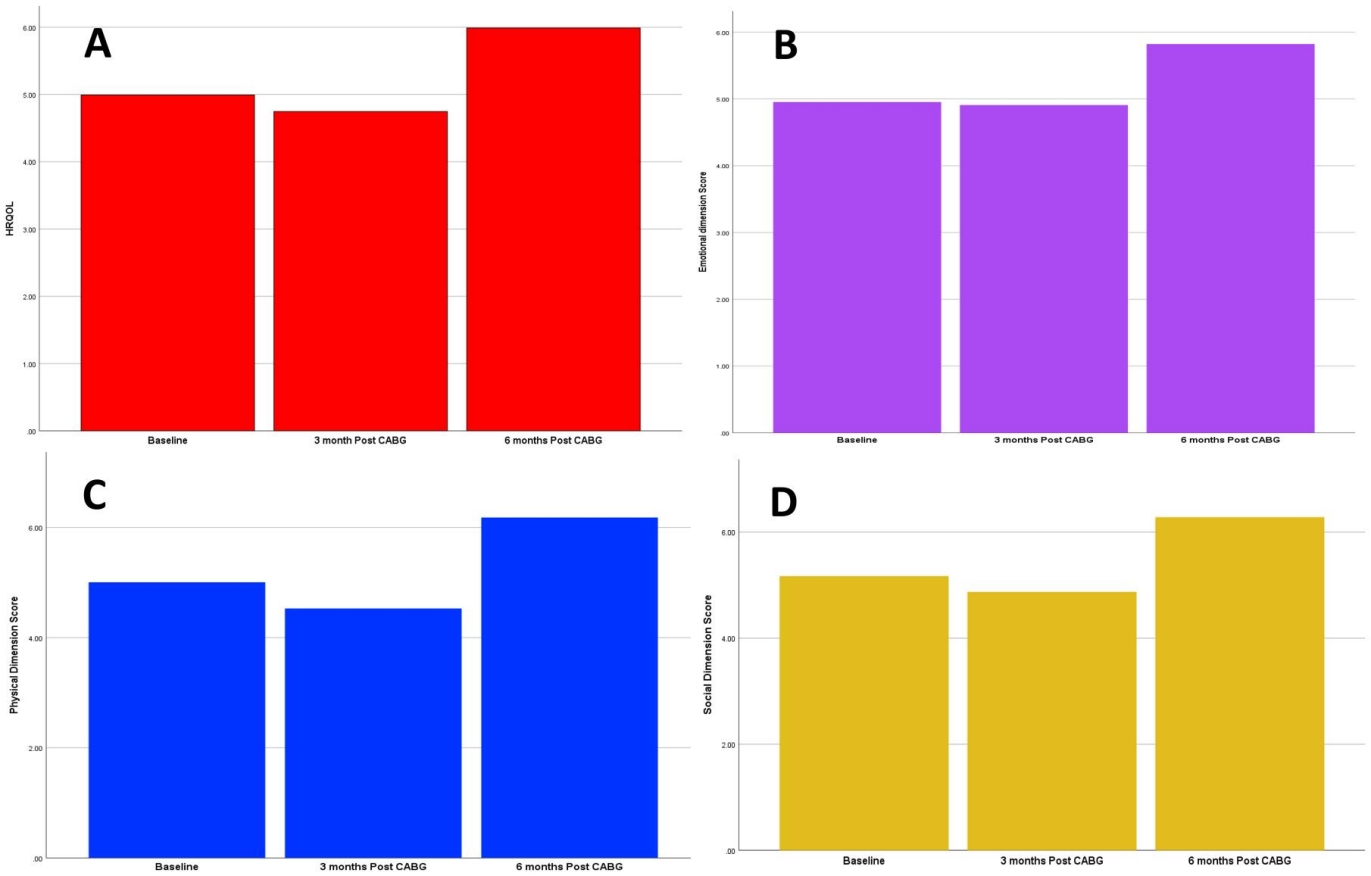
MacNew scores preoperatively, and at 3 and 6 months after surgery: (A) HRQOL total score, (B) emotional dimension, (C) physical dimension, and (D) social dimension

Social scores decreased from 5.21 ± 1.26 to 4.87 ± 1.24 at 3 months (*P* = .008) and rose to 6.28 ± 0.81 at 6 months (*P* < .001), surpassing the clinically relevant threshold. Overall, HRQOL and its domains showed a transient early decline followed by marked improvement above baseline at 6 months. These changes are illustrated in **Figure 1**.

### Age

Participants were categorized into 3 age groups (≤59, 60-69, ≥70 years) to ensure comparable group sizes and facilitate analysis. Age did not significantly influence HRQOL at any time point: preoperatively (*P* = .443), 3 months post-surgery (*P* = .401), or 6 months post-surgery (*P* = .526). Detailed HRQOL scores by age group are presented in **Table 2**.

**Table 2.** Associations Between HRQOL, Age, and Gender

HRQOL—age								
Time	Age	*n*	Mean	SD*	Range	Median	QI**, Q3*** IQR	*P*-value
Baseline	≤59	18	4.81	0.92	3.30-6.15	4.91	4.06-5.64	.443
							1.58	
	60-69	39	5.13	1.13	2.89-6.96	5.26	4.30-6.11	
							1.81	
	≥70	45	5.02	0.94	2.00-6.59	5.19	4.63-5.65	
							1.02	
3 Months post	≤59	18	4.53	0.90	2.37-6.00	4.73	4.01-5.04	.401
							1.03	
	60-69	37	4.89	1.04	2.26-6.52	4.96	4.17-5.82	
							1.65	
	≥70	42	4.71	1.02	2.52-6.30	4.91	4.00-5.44	
							1.44	
6 Months post	≤59	18	5.77	0.82	3.37-6.56	6.17	5.21-6.33	.526
							1.12	
	60-69	37	6.12	0.40	4.74-6.63	6.22	5.93-6.44	
							0.51	
	≥70	42	5.97	0.76	2.63-6.65	6.19	6.03-6.38	
							0.35	

HRQOL—gender								
Time	Gender	*n*	Mean	SD*	Range	Median	Q1, Q3*** IQR	*P*-value
Baseline	Male	80	5.13	0.98	2.89-6.96	5.26	4.42-5.94	.057
							1.52	
	Female	22	4.64	1.08	2.00-5.93	4.89	3.50-5.65	
							2.15	
3 Months post	Male	75	4.99	0.98	2.52-6.52	4.93	4.26-5.63	.011
							1.37	
	Female	22	4.23	0.94	2.26-5.41	4.52	3.68-5.01	
							1.33	
6 Months post	Male	75	6.06	0.61	2.63-6.56	6.26	6.04-6.41	.009
							0.37	
	Female	22	5.72	0.80	3.37-6.63	6.02	5.26-6.23	
							0.97	

Abbreviations: HRQOL, health-related quality of life; IQR, interquartile range; * SD, standard deviation; ** Q1, first quartile; ** Q3, third quartile; HRQOL, healthrelated quality of life; IQR, interquartile range.

### Gender

Preoperatively, men (median = 5.26, IQR = 4.41-5.94) had higher HRQOL scores than women (median = 4.89, IQR = 3.50-5.64), although this difference was not statistically significant (*P* = 0.057). At 3 months post-surgery, men demonstrated significantly higher HRQOL than women (*P* = .011), and this difference persisted at 6 months (*P* = .009). Detailed results by gender are presented in **Table 2**.

### Arrhythmias

No significant differences in HRQOL were observed between patients with and without postoperative arrhythmias at any time point: preoperatively (*P* = .704), 3 months post-surgery (*P* = .139), or 6 months post-surgery (*P* = .494) (**Table 3**). Atrial fibrillation was the most prevalent arrhythmia and showed no significant differences in HRQOL: preoperatively (*P* = .734), 3 months post-surgery (*P* = .253), or 6 months post-surgery (*P* = .948).

**Table 3.** Associations Between HRQOL-Arrhythmia and LVEF

HRQOL—arrhythmia								
Time	Arrhythmia	*n*	Mean	SD*	Range	Median	Q1**, Q3*** IQR	*P*-value
Baseline	Yes	27	4.98	1.10	2.96-6.96	5.00	4.19-6.00	.704
							1.81	
	No	75	5.04	0.99	2.00-6.59	5.22	4.44-5.78	
							1.34	
3 Months post	Yes	26	4.50	1.10	2.26-6.22	4.65	3.83-5.27	.139
							1.44	
	No	71	4.84	0.97	2.37-6.52	4.93	4.26-5.52	
							1.26	
6 Months post	Yes	26	5.91	0.83	2.63-6.56	6.17	5.87-6.37	.494
							0.5	
	No	71	6.02	0.60	3.37-6.63	6.19	5.93-6.41	
							0.48	

HRQOL—LVEF								
Time	LVEF	n	Mean	SD*	Range	Median	Q1**, Q3*** IQR	*P*-value
Baseline	≤40%	19	4.95	0.95	3.41-6.59	4.95	3.96-5.74	
							1.78	
	41-50%	46	5.24	0.87	2.89-6.96	5.26	4.72-5.89	.310
							1.17	
	>50%	37	4.80	1.18	2.00-6.52	4.96	3.86-5.67	
							1.81	
3 Months post	≤40%	17	4.43	0.85	2.37-5.56	4.70	3.83-5.02	
							1.19	
	41-50%	44	4.71	1.00	2.70-6.52	4.76	4.22-5.44	.289
							1.22	
	>50%	36	4.93	1.06	2.26-6.30	5.07	4.23-5.82	
							1.59	
6 Months post	≤40%	17	5.83	0.84	3.37-6.63	6.07	5.63-6.36	.529
							0.73	
	41%-50%	44	6.01	0.66	2.63-6.56	6.19	5.93-6.37	
							0.44	
	>50%	36	6.04	0.58	4.33-6.56	6.30	6.02-6.44	
							0.42	

Abbreviations: HRQOL, health-related quality of life; IQR, interquartile range; LVEF, left ventricular ejection fraction; * SD, standard deviation; ** Q1, first quartile; ** Q3, third quartile; HRQOL, healthrelated quality of life; IQR, interquartile range; LVEF, left ventricular ejection fraction.

### Left ventricular ejection fraction

Left ventricular ejection fraction category did not significantly influence HRQOL at any of the 3 assessment points: preoperatively (*P* = .310), 3 months post-CABG (*P* = .289), and 6 months post-CABG (*P* = .529). The associations between HRQOL, arrhythmias, and LVEF are summarized in **Table 3**.

### Type of surgery

No significant differences in HRQOL were observed between patients who underwent isolated CABG (*n* = 67) and those who had a combined procedure including CABG and valve surgery (*n* = 35) at any assessment time point: preoperatively (*P* = .313), 3 months post-surgery (*P* = .797), and 6 months post-surgery (*P* = .298). Non-significant differences observed in subgroup analyses (eg, gender and type of surgery) should be interpreted with caution, as they may reflect limited statistical power and the potential for type II error (**Table 4**).

**Table 4.** Associations Between HRQOL-Type of Surgery

HRQOL—type of surgery								
Time	Type	*n*	Mean	SD*	Range	Median	Q1**, Q3*** IQR	*P-*value
Baseline	CABG	67	5.10	1.00	2.96-6.96	5.26	4.30-5.96	.313
							1.66	
	CABG+Valve	35	4.89	1.04	2.00-6.52	5.04	4.44-5.67	
							1.23	
3 Months post	CABG	63	4.77	0.99	2.26-6.33	4.89	4.15-5.44	.313
							1.29	
	CABG+Valve	34	4.96	1.04	2.52-6.52	4.87	4.04-5.44	
							1.4	
6 Months post	CABG	63	5.93	0.75	2.63-6.63	6.15	5.85-6.37	.298
							0.52	
	CABG+Valve	34	6.30	0.48	4.74-6.56	6.10	5.93-6.41	
							0.48	

Abbreviations: CABG, coronary artery bypass grafting; HRQOL, health-related quality of life; IQR, interquartile range; Q1, first quartile; Q3, third quartile; SD, standard deviation.

## DISCUSSION

This study assessed HRQOL before and after CABG and explored differences by demographic and clinical factors over 6 months. Coronary artery bypass grafting not only prolongs survival but also improves functional capacity, independence, and quality of life.^4^^,^^22^ In this multicentre cohort, patients showed significant HRQOL improvement in all dimensions at 6 months.

The principal finding was a transient decline at 3 months (*P* = .015) followed by marked recovery at 6 months (*P* < .001). Similar patterns have been described in other prospective CABG studies.^5^^,^^11^^,^^19–21^^,^^23^ The temporary reduction likely reflects early postoperative recovery, when fatigue, dyspnoea, arrhythmias, renal dysfunction, or wound complications may limit physical and emotional well-being. As rehabilitation progresses, mobility and confidence typically return, which may explain the substantial HRQOL gains observed later. A recent meta-analysis confirmed sustained long-term HRQOL benefits after CABG.^4^ Structured cardiac rehabilitation and psychological support may help mitigate this early decline and facilitate smoother recovery trajectories.^5^

This study found significant HRQOL improvement after CABG in patients aged ≥70 years, with no differences compared to younger groups. These findings align with Peric et al,^15^ who reported substantial HRQOL gains in elderly patients 6 months postoperatively, and with another study showing comparable outcomes between older and younger cohorts.^9^ In contrast, one study reported lower QOL among elderly patients 1 year post-CABG.^16^ Such discrepancies underscore the need for further evaluation of CABG’s benefits and risks in older populations, as conclusions vary depending on age cut-offs and group definitions.^9^^,^^15^^,^^19^

Male gender was associated with greater HRQOL improvement, although both sexes showed significant postoperative gains. Men consistently reported higher scores, consistent with prior research linking female sex to lower HRQOL, poorer mental health, and slower recovery.^17^^,^^18^ These gender-related differences may stem from higher complication rates and less favourable psychological adaptation among women.^18^ The predominance of men in this cohort (78.4%) reflects the general CABG population.^18^ Despite this, women’s lower postoperative scores—especially in the physical domain—remain clinically relevant.^17^^,^^18^ Given the limited number of women and absence of multivariable adjustment, these observations should be interpreted cautiously. Larger, gender-balanced studies are needed to confirm these patterns and clarify underlying mechanisms.

Gender disparities may reflect both psycho-social and physiological mechanisms. Women undergoing CABG often report higher anxiety and depression, greater postoperative pain, and slower recovery than men.^10^^,^^17^ These differences may relate to comorbidities, hormonal status, and social support.

Gender-sensitive rehabilitation and psychological interventions may improve recovery and HRQOL outcomes in women.

Postoperative AF remains the most frequent arrhythmic complication after CABG, associated with increased morbidity and prolonged hospitalization.^24^ In this study, 27 of 102 patients (20.7%) developed arrhythmias, including 21 with AF, consistent with reported rates of 10%-45%.^25^ Neither arrhythmias nor AF were linked to HRQOL differences at any time point, likely due to effective management. Previous studies have shown mixed findings, with some identifying AF as a predictor of poorer HRQOL.^26^

Left ventricular ejection fraction is a key determinant of cardiac function and overall health.^1^ In this study, no association was found between LVEF and HRQOL at any time point. This contrasts with findings by Blokzijl et al,^6^ who identified preoperative LVEF as an independent risk factor for HRQOL decline. Other studies of patients with severely reduced LVEF (<35%) undergoing CABG have demonstrated postoperative improvements in both HRQOL and LVEF.^27^ These observations indicate that CABG can be beneficial for HRQOL even in patients with significant left ventricular dysfunction.

No significant HRQOL differences were observed between isolated CABG and combined CABG with valve surgery at any time point. Preoperative scores were slightly higher in the isolated group, while postoperative scores were marginally lower, without statistical significance. Both groups improved postoperatively, consistent with reports showing HRQOL benefits across CABG, AVR, MVR, and combined procedures.^28^^,^^29^ In contrast, Grazulyte et al^30^ found greater health gains with combined CABG and valve surgery. Further studies are needed to clarify the influence of combined procedures on specific HRQOL domains.

Overall, CABG was associated with significant HRQOL improvement up to 6 months postoperatively, irrespective of age, LVEF, presence of AF, or surgical type. The identification of gender-related differences highlights the need for tailored strategies, particularly for women, to optimize recovery and quality of life. These findings can inform individualized rehabilitation programs.

Future research should include longer follow-up, examine the mechanisms of gender disparities, and explore HRQOL subdomains to better understand psycho-social recovery after CABG.

### Limitations

This study has several limitations. Reliance on self-reported medical histories may have introduced recall bias. The exclusion of emergency cases and use of convenience sampling limit generalizability. Although primarily focused on CABG, some participants underwent combined valve surgery, adding heterogeneity but reflecting real-world practice. The absence of systematic symptom classification (eg, NYHA or CCS class) limits the ability to fully characterize baseline functional severity. The follow-up period of 6 months does not allow assessment of longer-term HRQOL outcomes. The limited number of female participants and inclusion of both isolated and combined CABG cases may have contributed to sample imbalance; although no significant HRQOL differences were detected, larger studies with balanced gender representation are needed to confirm these findings. The use of univariate non-parametric tests may not fully adjust for confounding factors such as age, comorbidities, or baseline LVEF. Multivariable or mixed-effects modelling was not feasible due to the modest sample size, and subgroup comparisons were underpowered, so type II error cannot be excluded.

## Data Availability

The datasets generated and/or analysed during the current study are not publicly available due to patient confidentiality but are available from the corresponding author on reasonable request.

## ABBREVIATIONS

AF: Atrial fibrillation
CABG: Coronary artery bypass grafting
CAD: Coronary artery disease
HRQOL: Health-related quality of life
LVEF: Left ventricular ejection fraction
SD: Standard deviation
SPSS: Statistical Package for the Social Sciences
